# Comprehensive approach to the management of the patient with multiple rib fractures: a review and introduction of a bundled rib fracture management protocol

**DOI:** 10.1136/tsaco-2016-000064

**Published:** 2017-01-05

**Authors:** Cordelie E Witt, Eileen M Bulger

**Affiliations:** Department of Surgery, University of Washington, Seattle, Washington, USA

**Keywords:** rib fractures, morbidity and mortality, pain management, protocols

## Abstract

Rib fractures are common among patients sustaining blunt trauma, and are markers of severe bodily and solid organ injury. They are associated with high morbidity and mortality, including multiple pulmonary complications, and can lead to chronic pain and disability. Clinical and radiographic scoring systems have been developed at several institutions to predict risk of complications. Clinical strategies to reduce morbidity have been studied, including multimodal pain management, catheter-based analgesia, pulmonary hygiene, and operative stabilization. In this article, we review risk factors for morbidity and complications, intervention strategies, and discuss experience with bundled clinical pathways for rib fractures. In addition, we introduce the multidisciplinary rib fracture management protocol used at our level I trauma center.

## Introduction

### Fracture patterns and clinical outcomes

Rib fractures occur in ∼9–10% of patients with trauma,^1^ ^2^ and are markers of severe bodily and solid organ injury. In addition to being associated with concomitant thoracic injuries, rib fractures are associated with head, extremity, abdominal, and blunt cardiac injuries.^1–4^ These patients have poor overall clinical outcomes, and are at increased risk for multiple complications. Thoracic complications of rib fractures are common, including pneumonia, pulmonary effusion, aspiration, acute respiratory distress syndrome (ARDS), pulmonary emboli, and atelectasis or lobar collapse;^1^ ^4^ these are important as targeted interventions may enable risk reductions. An evaluation of the National Trauma Data Bank (NTDB) from 1994 to 2003 identified a mortality rate of 10% and a 13% complication rate, of which 48% were pulmonary complications.^2^ Among 711 patients at a level II trauma center, 45% of patients with rib fracture experienced complications, including 35% sustaining pulmonary complications.^1^ In addition, 34% were discharged to a long-term care facility and 12% died; pulmonary complications accounted for 54% of late deaths within this population.^1^

The number of rib fractures sustained correlates with mortality and complication risk in multiple studies. In the 1994–2003 NTDB study described above, patients with single rib fractures had 5.8% mortality, 5 fractured ribs conferred 10% mortality, 6 with 11.4%, 7 with 15%, and 8 or more with 34.4% mortality. Likewise, additional rib fractures were associated with increased risk of death, pneumonia, ARDS, pneumothorax, aspiration pneumonia, empyema, intensive care unit (ICU) length of stay (LOS), and hospital LOS.^2^ The level II trauma center study reported 5% mortality among patients with 1–2 ribs fractured, and 29% mortality among those with 7 or more fractures; 52% of their rib fracture patients overall required ICU-level care; additionally, number of rib fractures correlated with mortality, hypotension, and Injury Severity Score (ISS).^1^ However, a study from the NTDB showed that number of fractures correlated with mortality on univariate but not multivariable analyses;^5^ and a study of 594 patients from one institution found that number of rib fractures was not associated with mortality.^6^ However, a meta-analysis showed that higher numbers of rib fractures was associated with mortality, with a combined odds ratio (OR) of 2.02 (95% CI 1.89 to 2.15) for death among patients with three or more rib fractures compared with those with fewer rib fractures.^7^

Rib fracture location and pattern is clinically important. Presence of bilateral fractures and concomitant lung parenchymal injuries were associated with increased risk of chest-related death in a cohort of 1495 patients from one institution.^8^ Among another cohort of 1490 patients with blunt chest trauma, mortality was associated with presence of flail chest in addition to number of fractures.^9^ Flail chest alone is associated with mortality of 16–17%.^9–11^ Mortality risk increased to 42% when pulmonary contusion was present in addition to flail.^10^ Presence of a first rib fracture was associated with 36% mortality in one study and high likelihood of concomitant injuries.^12^

### Scoring systems

Clinical and radiographic scoring systems have been developed for patients with thoracic trauma and rib fractures for risk assessment. Pape *et al* 8 reported a scoring system with an area under the receiver operating characteristic curve of 0.916–0.924 for chest-related complications or death based on number of rib fractures, arterial oxygen tension:fractional inspired oxygen ratio, presence of lung contusions, pleural involvement and patient age. Chapman and colleagues established RibScore, a radiographic scoring system assessing presence of ≥6 rib fractures, bilateral fractures, flail chest, ≥3 severely displaced fractures, first rib fracture, and presence of fractures in the anterior, lateral and posterior regions. This scoring system was associated with development of pneumonia, acute respiratory failure, and tracheostomy with areas under the receiver operating characteristic curves of 0.71–0.75.^3^ Patients who developed pneumonia, acute respiratory failure, or who required tracheostomy had higher summed RibScores than those who did not develop such complications. Likewise, bivariate analyses showed that each of the variables in the RibScore correlated with the same complications.^3^

### Long-term outcomes

With regard to long-term outcomes, Marasco and colleagues assessed patients with trauma admitted to their institution with multiple rib fractures, including patients with isolated thoracic injuries as well as patients with polytrauma. They found that at 6 months, only 36% of isolated thoracic injury and 23% of patients with polytrauma had Glasgow Outcome Score Extended (GOS-E) scores of 7–8, and at 24 months only 44% of isolated thoracic injury patients and 30% of patients with polytrauma had GOS-E scores of 7–8.^13^ The GOS-E is an interview-based functional assessment scored from 1 to 8, where 7 and 8 indicate good recovery (upper and lower), 5 and 6 indicate moderate disability (upper and lower), 3 and 4 indicate severe disability (upper and lower), 2 indicates vegetative state, and 1 indicates death.^14^ ^15^ Patients scoring 7 have returned to preinjury work status though may have impairments in social and extracurricular activities; patients scoring 8 have minimal to no sequelae from their injury affecting their daily lives.

A prospective study following 203 individuals at one institution showed similar trends toward long-term pain and disability following rib fractures. One hundred and eighty-seven of the 203 enrolled patients were followed to 2 months postinjury, at which time 76% had persistent disability defined based on decreased work or functional status, and 59% had ongoing chest pain based on McGill Pain Questionnaire and Present Pain Intensity scores.^16^ One hundred and sixty-one of the 203 enrolled patients were followed to 6 months postinjury, at which time 53% had chronic disability and 22% had chronic pain. Of 89 patients with isolated rib fractures, 40% had chronic disability and 28% had chronic pain.^17^

### Elderly patients

Elderly patients have been consistently shown to have poorer outcomes following rib fractures, which may relate to comorbidities, reduced physiological reserve, and greater difficulty assessing and managing hemodynamics.^4^ Meta-analysis showed that patients aged ≥65 had a combined OR for mortality of 1.98 compared with younger patients,^9^ with low heterogeneity.^7^ A study using Taiwan's National Health Insurance Research Database over 2002 through 2004 evaluated 18 856 patients with rib fractures from motor vehicle crashes and found that age was associated with increased crude and adjusted risk of death within 24 hours;^18^ a 13-year study of 27 855 patients with trauma in Pennsylvania with multiple rib fractures found that patients aged ≥65 had mortality of 20.1% compared with 11.4% in younger patients, including when stratified by number of ribs fractured.^19^ Elderly patients in this study had longer hospitalizations, longer ICU lengths of stay, longer ventilator requirements, and greater risk of acute respiratory failure, pneumonia, and pulmonary effusion.^19^ Likewise, a retrospective cohort of patients treated at a level I trauma center over 10 years showed that patients ≥65 years had longer mean ventilator days, ICU days, and hospital stay; mortality was two times higher in elderly patients and increased with number of rib fractures.^4^ Regarding complications, this study showed that pneumonia occurred in 31% of elderly compared with 17% of young patients, late pulmonary effusion occurred in 41% of elderly and 14% of young, ARDS occurred in 9% of elderly and 5% of young, and lobar collapse occurred in 8% of elderly and 3% of young patients. Elderly patients with 3–4 rib fractures had a 19% mortality rate and 31% frequency of pneumonia; those with more than 6 rib fractures had 33% mortality and 51% frequency of pneumonia.

## Evidence-based therapeutic interventions

Given the multiple complications associated with rib fractures, several evidence-based strategies have been advocated for risk mitigation. Appropriate analgesia and early aggressive care appears to attenuate the development of pulmonary complications^4^ ^20^ ^21^ through enhancement of patients' functional capacity such as reducing splinting and improving pulmonary function.

### Catheter-based analgesia

Catheter-based analgesia, including epidural and paravertebral nerve catheters, may be beneficial in patients with multiple rib fractures. Epidural analgesia has been associated with lower mortality in elderly and young adult patients,^2^ ^4^ ^22^ and there is thought that it could reduce complications through improved clearance of secretions, maintenance of pulmonary function, reduced atelectasis from splinting, and greater ease in weaning from the ventilator.^2^ ^4^ A recent joint practice management guideline from the Eastern Association for the Surgery of Trauma (EAST) and Trauma Anesthesiology Society provided recommendations regarding catheter-based analgesia in adult patients with blunt thoracic trauma. Epidural analgesia was conditionally recommended over non-regional modalities given, although the quality of evidence was found to be poor.^23^ They found that the included studies were mixed as to findings regarding mortality benefit, and meta-analysis showed no significant difference. Assessed studies included a randomized trial with 46 patients randomized to receive epidural versus systemic opioids, where patients in the epidural group had improved pain control, lower frequency of pneumonia, shorter ventilator duration, and similar ICU LOS and mortality.^24^ Another assessed study using data from the National Study on Cost and Outcomes of Trauma database found that patients who received epidural catheters were more likely to be treated in trauma centers, had lower odds of death at 30, 90, and 365 days compared with propensity-matched controls.^22^

In addition, there was no significant difference in postoperative pulmonary complications on the EAST guidelines' meta-analysis.^23^ In pooled data, they found that epidural analgesia was associated with lower pain scores at 24 and 48 hours, but not at 72 hours; in addition, epidural was associated with fewer ventilator days albeit higher odds of requiring mechanical ventilation, shorter ICU LOS, and no reduction in overall hospital LOS. Overall, there was limited data from randomized controlled trials, variable study quality, and heterogeneous data. The authors note that specific populations may derive greater benefit.^23^

Although these data suggest that epidural may be beneficial in patients with multiple rib fractures, patients with multisystem trauma may have contraindications. Epidural-related sympathetic blockade with anesthetic agents may lead to hypotension,^25^ which can be problematic in patients with unstable hemodynamics and/or competing injuries. In addition, patients with rib fractures may have concomitant thoracic spine injury, which present a technical contraindication to epidural placement.^26^ Low molecular weight heparin is the recommended thromboembolic prophylactic agent in most patients with multisystem trauma, given high risk of thromboembolic complications;^27^ ^28^ however, is not recommended while epidural catheters are inserted or removed, given risk for epidural hematoma development.^28–30^ Patients with thrombocytopenia or coagulopathy may be similarly inappropriate candidates.^26^ Ultimately, thoughtful consideration of prophylactic timing, concomitant trauma and individual patient risk factors is warranted.

In the context of these potential contraindications, other regional anesthesia techniques have been considered. The EAST guidelines also assessed paravertebral nerve blockade, continuous intrapleural infusions and continuous intercostal infusions but found insufficient studies to make recommendations.^23^ In one study, which compared paravertebral nerve block to epidural among 30 patients, there was no significant difference in pain control between groups. Frequency of complications and hospital LOS was similar in the two groups.^31^

A previous EAST Practice Management Guidelines Work Group published pain management guidelines for blunt thoracic trauma, providing level I recommendations in favor of epidural over narcotic analgesia to reduce subjective pain perception as well as improvement in pulmonary function, suggesting that epidural should be the preferred technique after blunt thoracic trauma. Level II recommendations suggested epidural for patients with ≥4 rib fractures or age ≥65 unless contraindicated, as well as for younger patients if not contraindicated. Additionally, there was level II data to support decreased ventilator days, ICU LOS and hospital LOS with epidural compared with narcotic, and regarding the utility of paravertebral or extrapleural infusions to improve pain and possibly pulmonary function.^21^

### Multimodal pain therapy

Multimodal systemic analgesia, which combines opioids with non-opioid adjuncts, has been advocated in some studies albeit data are also limited. The recent EAST and Trauma Anesthesiology Society guidelines make a conditional recommendation in favor of multimodal therapy, given some data on improved analgesia and in the setting of known side effects of opioids.^23^ Administration of intravenous (IV) ibuprofen to patients with traumatic rib fractures was associated with lower opioid requirement and improved pain scores compared with patients who did not receive intravenous ibuprofen in a small study.^32^ In a retrospective study of patients with multiple rib fractures receiving ketorolac versus patients who did not, ketorolac was associated with decreased frequency of pneumonia, more ventilator-free days, and more ICU-free days. Complications from ketorolac use were uncommon in their cohort.^33^

Multimodal therapy has been advocated following ambulatory surgery^34^ and cardiac surgery via sternotomy.^35^ A study of 86 patients requiring thoracotomy for lobectomy showed that administration of parecoxib compared with placebo, in addition to patient-controlled thoracic epidural, was associated with improved pain scores and lower serum cortisol and adrenocorticotropic hormone (ACTH) levels.^36^

Data regarding gabapentin are more common; however, most studies have assessed preoperative use. In a prospective, randomized study of elective thoracotomy patients receiving a single 600 mg dose of preoperative gabapentin versus placebo as an adjunct to standard epidural, ketorolac, acetaminophen, and IV patient-controlled analgesia, there was no difference between groups for pain scores, nausea/vomiting, respiratory depression, or chronic pain 3 months postsurgery.^37^ In contrast, a meta-analysis of preoperative gabapentin administration (as well as postoperative gabapentin in some studies) among surgical patients (abdominal, spine, orthopedic, gynecological, breast, and ear, nose, and throat) showed improved pain scores and reduced 24-hour opioid consumption among gabapentin recipients compared with control.^38^ Another meta-analysis of surgical patients (abdominal, orthopedic, gynecological, thyroid, breast, prostatectomy, caesarean section and thoracotomy) showed preoperative gabapentin to be associated with lower postoperative opioid use, though increase in postoperative somnolence.^39^ Thus, although there are limited data regarding multimodal therapy in patients with rib fractures specifically, data in these other settings support its potential utility.

### Pulmonary hygiene

Pulmonary hygiene methods have been advocated to reduce the development of complications following rib fractures or thoracic trauma.^20^ Incentive spirometry is an easily implemented measure, and can be guided by bedside nurses.^20^ Bakhos and colleagues reported that in 38 patients aged ≥65 with rib fractures from blunt chest trauma, bedside measurement of vital capacity by a respiratory therapist within 48 hours of admission correlated with hospital LOS, ICU LOS, and discharge to extended care facility, although no correlation with subsequent development of pulmonary complications in this small sample. In an emergency department setting, spirometry-derived inspiratory capacity was lower among patients with torso injury, although patient outcomes were not assessed.^40^

### Operative stabilization

Surgical stabilization may be indicated for some patients with severe rib fractures, although there are no published practice management guidelines relating to this topic at present.

In the setting of flail chest, two meta-analyses have shown benefit to surgical fixation on pooled analyses. The first identified 11 studies comparing fixation to non-operative management of which two were randomized trials; pooled analysis showed that surgical fixation was associated with shorter ventilator duration, ICU LOS, and hospital LOS, as well as reduced odds of pneumonia, septicemia, tracheostomy, chest deformity, and even mortality.^41^ The second meta-analysis included nine studies, of which seven were also in the prior study. They drew similar conclusions, finding that fixation was associated with shorter ventilator duration, ICU LOS, and hospital LOS, as well as decreased risk of pneumonia, tracheostomy, and mortality.^42^ Still, surgical fixation of flail chest is uncommon nationwide: a study from the NTDB found that just 0.7% of 3467 adults with flail chest from blunt trauma who were treated at levels I and II trauma centers underwent surgical fixation.^11^ An additional prospective randomized controlled trial published after these meta-analyses studied 23 patients treated operatively and 23 treated non-operatively in Australia; they observed that the operative group had shorter ICU LOS and lower risk for receiving noninvasive ventilation after extubation, or receiving tracheostomy. There was a trend toward lower frequency of pneumonia, and no significant difference in failure of extubation, ICU readmission, hospital LOS, or mortality. Quality of life assessment at 6 months demonstrated no difference in Short Form-36 items. Cost analysis demonstrated savings among the operatively managed group given the reduction in ICU needs, despite the cost of the operation.^43^

Among patients with multiple rib fractures without flail, several studies have reported successful institutional use of the surgical stabilization with encouraging outcomes;^44–47^ however, prospective studies are needed.

## Bundled clinical care pathways

Standardized clinical pathways have been used across a variety of disciplines to streamline and improve care. Several have been presented in the literature which include or focus on patients with multiple rib fractures.

Sesperez *et al* 48 reported on implementation of several trauma-related clinical pathways, including one for patients with rib or sternal fractures, at their institution in 1998–1999. While protocol details were not provided, the authors state that this and the other four trauma protocols (severe head injury, pelvic fracture, blunt abdominal trauma, femur fracture) included assessment, treatment, investigations, nutrition/hydration, elimination, pain management, medications, activity, skin integrity, physiotherapy, consultation, education, and patient discharge. Pathway adherence was assessed based on number of observed versus expected variance over the course of implementation; outcome achievement for the rib fracture pathway was 93–96% across the timeframe of the study indicating feasibility. Patient outcomes were not assessed; however, subsequent studies of other rib fracture clinical pathways do include outcomes data.

Sahr and colleagues reported on an emergency department (ED) triage protocol for patients ages ≥65 evaluated at their level I trauma center, such that patients with ≥3 rib fractures, hemodynamic abnormality or hemothorax were automatically referred to the trauma service, as well as those sustaining falls from higher than ground level or with recent use of anticoagulant or antiplatelet drugs. These patients who were automatically referred to the trauma service, compared with patients meeting the same clinical criteria preprotocol, had shorter hospital LOS and ICU LOS on univariate analysis.^49^ While this study evaluated a small cohort and had the inherent limitations of a before–after study, it supports the concept that a simple process-focused intervention may improve outcomes.

Todd *et al* 50 reported on their multidisciplinary clinical pathway at their level I trauma center, enrolling patients aged >45 with ≥4 rib fractures, excluding patients with Glasgow Coma Score (GCS) <8 and abnormal brain CT. Eligible patients were admitted to monitored beds, were taught incentive spirometry, and received patient-controlled analgesia. During the first 3 days, patients' pain (scored 1–10), inspiratory volume, and cough were evaluated by nursing staff. Patients pain score >6, incentive spirometry volume <15 mL/kg, or weak cough were entered into a pathway with respiratory therapy interventions, pain service consultation (with consideration of epidural analgesia or other modes), physical therapy for strength and mobility, and nutritional optimization. Institutional trauma registry data were used to analyze patients, injuries, and outcomes after pathway implementation as compared with a historical control prior to implementation. The authors found that after implementation, patient-controlled analgesia was used more often and there was greater usage of epidural catheters. Multivariate analysis showed that postpathway implementation patients had significantly decreased ICU LOS, hospital LOS, pneumonia, and mortality.^50^

Ultimately, these studies suggest that interventions focusing on processes-of-care for patients with rib fractures are feasible and have potential to improve patient outcomes.

## Harborview Medical Center rib fracture management protocol

Harborview Medical Center is a state designated level I adult and pediatric trauma center, serving Washington, Alaska, Montana, and Idaho. The facility cares for ∼4000–4500 patients with blunt trauma annually, of which ∼600 per year have at least three rib fractures. Our facility has adopted a multidisciplinary bundled care pathway for management of patients with multiple rib fractures, which will be presented in this section. It incorporates many of the tenets proposed by Todd *et al*, but with broader inclusion criteria, early initiation of multimodal pain therapy, and frequent function-based scoring driven by nursing staff and the patient.

**Figure 1:**
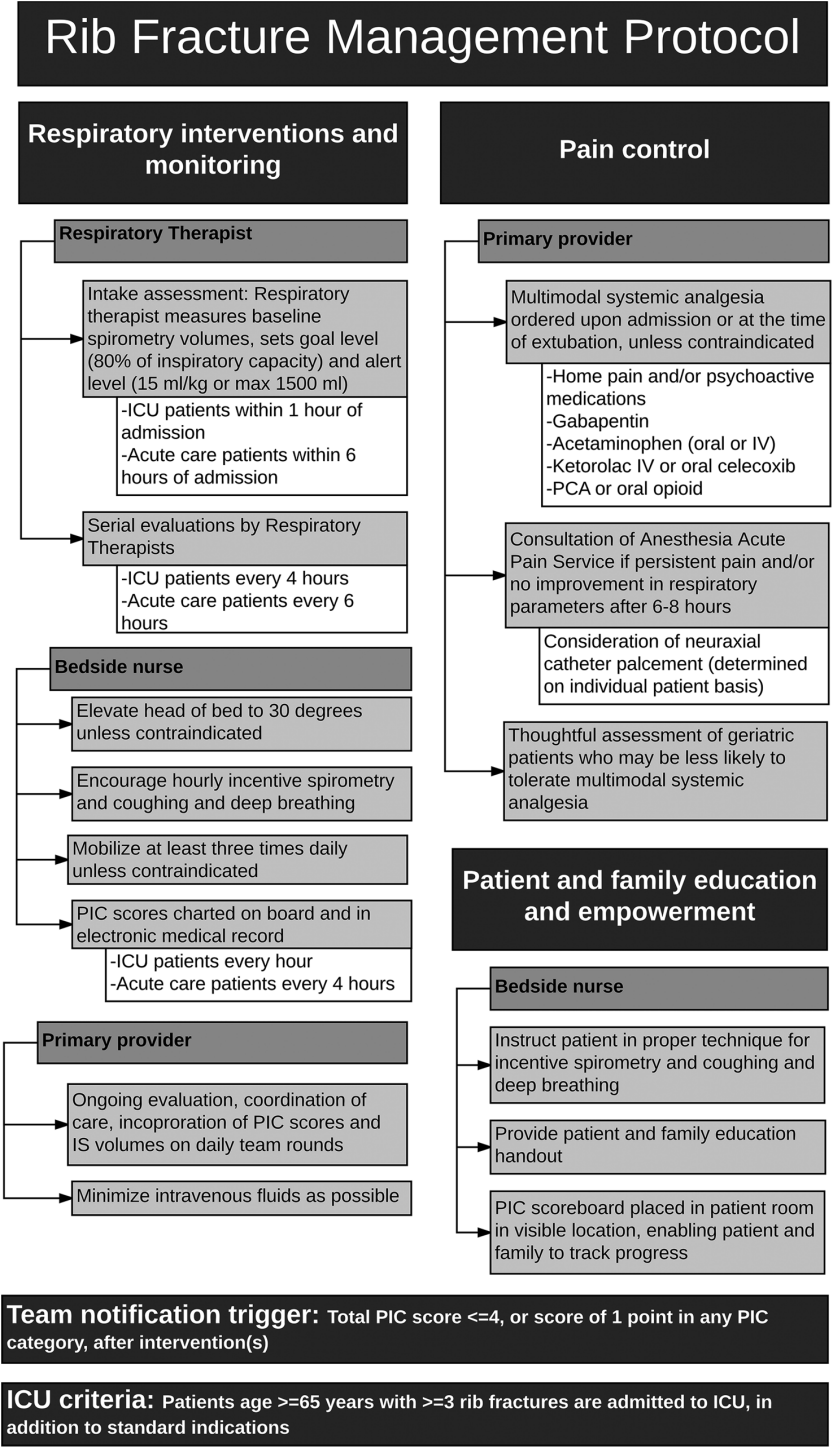
Harborview Medical Center rib fracture management protocol. ICU, intensive care unit; IS, incentive spirometry; IV, intravenous; PIC, Pain, Inspiratory capacity, and Cough; PCA, patient-controlled analgesia.

The Harborview rib fracture management protocol applies to all patients admitted with acute rib and/or sternal fracture(s) who meet the following criteria: age >14 years, extubated or recently extubated, GCS 13–15, and absence of high spinal cord injury. Patients over age 65 with ≥3 rib fractures are to be admitted to the ICU. The protocol includes interventions and monitoring by nursing staff, respiratory therapists, and physicians, and includes specific guidelines for ICU and acute care status patients, as further depicted in figure 1.

### PIC scoring tool

**Figure 2:**
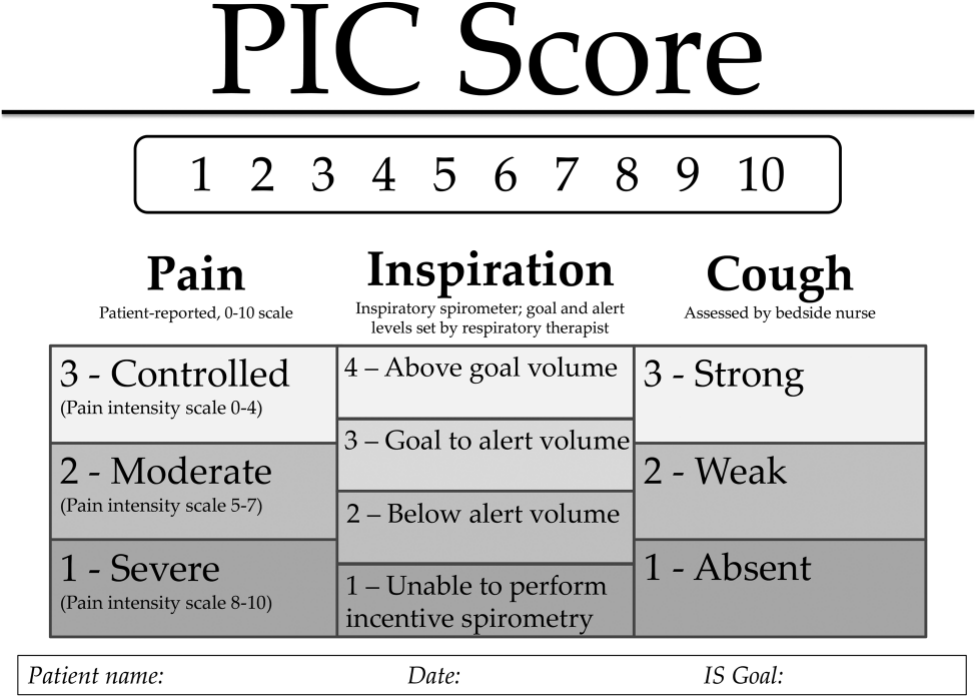
Harborview Medical Center PIC scoreboard. IS, incentive spirometry; PIC, Pain, Inspiratory capacity, and Cough.

The pathway uses a PIC scoring tool to serially evaluate and monitor patients, referring to *P*ain, *I*nspiratory capacity, and *C*ough, as shown in figure 2. This score was originally developed by Wellspan York Hospital, York, Pennsylvania, USA and presented at the Trauma Quality Improvement Project meeting in 2014. We adopted this score with their permission. The composite score may range from 3 to 10 where 10 is the goal score. Pain is scored on a scale of 1–3, representing patient-reported pain score on the subjective 0–10 scale: 3 points if controlled (subjective numeric scale 0–4), 2 points if moderately controlled (subjective numeric scale 5–7), or 1 point if severe (subjective numeric scale 8–10). Inspiratory capacity is scored on a scale of 1–4, relating to ‘goal’ and ‘alert’ levels for inspiratory spirometry based on sex-specific predictive normograms for age and height as available in the spirometer product inserts (goal is set at 80% of expected inspiratory capacity, alert level is 15 mL/kg or a maximum of 1500 mL). Patients receive four points if able to achieve at least goal inspiratory spirometry volume, three if between goal and alert levels, two if less than alert volume, and one point if unable to perform inspiratory spirometry. Finally, cough is subjectively assessed by the bedside nurse and assigned three points if strong, two points if weak, and one point if absent.

PIC scores are explained to the patient and family, and charted in a visible location in the patient's room as well as in the electronic medical record. Patients receiving ICU care undergo hourly assessment of PIC score while awake, and patients receiving acute floor-level care undergo assessment every 4 hours. The responsible physician and respiratory therapist are notified if a patient receives a score of 1 in any category or an overall score ≤4 despite interventions.

### Multimodal systemic analgesia

Under the rib fracture management protocol, patients receive multimodal systemic pain management on admission. Unless contraindications apply, patients should receive any home pain medications (including psychoactive medications and opioids), oral gabapentin (dose adjusted for age and renal function when indicated), oral or IV acetaminophen (except in geriatric patients or those with liver dysfunction), and either IV ketorolac or oral celecoxib. In addition, patients are prescribed oral opioids or patient-controlled opioid analgesia, titrated to effect or side effects.

When initial multimodal systemic analgesia is ineffective, our institution's Acute Pain Service is consulted for consideration of neuraxial catheter placement.

### Nursing, respiratory therapy, and physician components

The primary physician places electronic orders for the protocol with admission orders, or at the time of extubation if patients are initially intubated. This includes hourly pulmonary hygiene, minimization of IV fluids as possible, frequent mobilization, elevation of head of bed, respiratory therapy, and multimodal analgesia. PIC scores and spirometry volumes are incorporated into daily team rounds, and are used to guide care decisions.

Nursing-based interventions include assessment and charting of PIC scores, patient and family education, encouragement of frequent pulmonary hygiene and early mobilization. A patient and family education brochure is provided and family members are encouraged to support the patient in incentive spirometry use to improve their scores.

Respiratory therapists evaluate all ICU patients within 1 hour of admission, and all acute care floor patients within 6 hours of admission. Therapists set the goal and alert inspiratory spirometry levels as described above, and perform frequent monitoring and care. ICU patients on the protocol receive respiratory therapy every 4 hours; acute care patients receive therapy every 6 hours.

For patients without improved pain scores and respiratory parameters despite 6–8 hours of multimodal analgesia, our hospital's Anesthesiology Acute Pain Service is consulted for consideration of neuraxial catheter placement. Decision for neuraxial catheter placement is made on an individual patient basis, with consideration to pain level, respiratory capacity, coagulation status, mental status, spine injuries and positioning limitations, sepsis or infection, allergies, comorbidities and hemodynamics.

Early surgical rib stabilization (within 72 hours following trauma admission) is considered for patients meeting any of the following anatomical criteria: 3 or more displaced rib fractures, flail segment, patients who require video-assisted thoracoscopic surgery (VATS) or thoracotomy, or patients who have marked sternal flail and overlapping sternal fractures. Likewise, surgical rib stabilization is considered for patients who fail extubation attempts due to mechanical instability or pain.

## Summary

Rib fractures are important indicators of injury severity, and patients with multiple rib fractures carry high risk of morbidity and mortality. Based on the existing literature, we recommend a comprehensive and standardized management algorithm, such as our institutional bundled care pathway presented here, including consideration of catheter-based analgesia, multimodal pain management, respiratory therapy interventions, and frequent reevaluation.
